# Comparative evaluation of bonding failure rate with two light cure orthodontic adhesives: a cross-arch split-mouth prospective study

**DOI:** 10.1590/2177-6709.30.2.e2524214.oar

**Published:** 2025-05-23

**Authors:** Dhruv JAIN, Sukhbir Singh CHOPRA, Vivek Kumar THAKUR, Surendra Kumar SEWDA

**Affiliations:** 1 Army Dental Centre (Research & Referral), Department of Orthodontics & Dentofacial Orthopedics (New Delhi, India).

**Keywords:** Orthodontic bonding, Orthodontic adhesive, Bond strength, Bond failure rate, Bracket survival time, Colagem ortodôntica, Adesivo ortodôntico, Resistência da colagem, Taxa de falhas de colagem, Tempo de sobrevivência do braquete

## Abstract

**Introduction:**

Continuous improvements in the field of adhesive dentistry have led to the introduction of various materials that have the potential to be used as orthodontic adhesives. One such light-cure orthodontic adhesive is the U-Bond™ Ortho, which clinically exhibits optimum bonding strength.

**Objective:**

The aim of this cross-arch split-mouth prospective study was to evaluate the bonding failure rate along with the bracket survival time of U-Bond™ Ortho as compared with Transbond™ XT adhesive material, over an observation period of six months.

**Material and methods:**

36 consecutive patients (16 males and 20 females; mean age 22.44 years) fulfilling the selection criteria were included in the study. A total of 720 brackets were bonded equally using Transbond™ XT and U-Bond™ adhesive, which were analyzed for bracket failure rate, using the Chi-square test or Fischer test, and mean bracket survival time using Kaplan-Meier analysis.

**Results:**

The overall bracket failure rate was 3.6% (2.8% and 4.4% for Transbond™ XT and U-Bond™ respectively). The results were statistically non-significant when both adhesive materials were compared for differences in bonding failure rate and mean bracket survival time in the maxillary and mandibular arches, and anterior and posterior segments, although gender-based differences were statistically significant.

**Conclusion:**

Both Transbond™ XT and U-Bond™ Ortho exhibited similar clinically acceptable performances in terms of bonding failure rate and bracket survival time.

## RESUMO

**Introdução:**

Melhorias contínuas no campo da adesão em Odontologia levaram à introdução de novos materiais que têm o potencial de serem usados como adesivos ortodônticos. Um desses adesivos ortodônticos fotopolimerizáveis é o U-Bond™ Ortho, que clinicamente apresenta uma ótima força de adesão.

**Objetivo:**

O objetivo desse estudo prospectivo de boca dividida foi avaliar a taxa de falhas de colagem e o tempo de sobrevivência de braquetes colados com U-Bond™ Ortho, em comparação com o adesivo Transbond™ XT, em um período de observação de seis meses.

**Material e métodos:**

36 pacientes consecutivos (16 homens e 20 mulheres; idade média de 22,44 anos) que preencheram os critérios de seleção foram incluídos no estudo. No total, 720 braquetes foram colados usando Transbond™ XT ou U-Bond™, e foram analisados quanto à taxa de falhas de colagem, usando o teste do qui-quadrado ou o teste de Fischer, e ao tempo médio de sobrevivência do braquete, usando a análise de Kaplan-Meier.

**Resultados:**

A taxa geral de falhas dos braquetes foi de 3,6% (2,8% e 4,4% para Transbond™ XT e U-Bond™, respectivamente). Os resultados não foram estatisticamente significativos quando ambos os materiais adesivos foram comparados quanto às diferenças na taxa de falhas de colagem e no tempo médio de sobrevivência dos braquetes nas arcadas superior e inferior e nos segmentos anterior e posterior, embora as diferenças relativas ao sexo tenham sido estatisticamente significativas.

**Conclusão:**

Tanto o Transbond™ XT quanto o U-Bond™ Ortho apresentaram desempenhos clinicamente aceitáveis e semelhantes em termos de taxa de falhas de colagem e tempo de sobrevivência do braquete.

## INTRODUCTION

Orthodontic bonding has become an integral aspect of fixed orthodontic mechanotherapy since the introduction of the acid-etching technique by Buonocore^1^ in 1955. The clinical success of bonding procedures depends largely on the mechanical and chemical characteristics of the orthodontic adhesive. Orthodontic bonding adhesives are specifically formulated to provide optimum bonding strength to withstand the orthodontic and masticatory forces during treatment while minimizing enamel damage during debonding procedures.^2^

Transbond™ XT (3M Unitek, USA), a time-tested light-cure orthodontic adhesive, offers excellent colour stability, low film thickness, extended working time and superior handling characteristics, making it a preferred choice for many orthodontists.^3^ However, its high cost may be considered a limiting factor. U-Bond™ Ortho (Vericom, Korea) is a recently introduced low-cost light cure orthodontic adhesive, with characteristic colour changing property following light cure, that provides for easy removal of residual adhesive.^4^ The differences in the mechano-chemical nature of the two adhesives is primarily due to the presence of bisphenol-A-glycidyl methacrylate (Bis-GMA) and triethylene glycol dimethacrylate (TEGDMA) along with silica fillers in Transbond™ XT, in comparison to the presence of proprietary blend of dimethacrylates in U-Bond™ Ortho. 

As a recently introduced orthodontic adhesive, the scientific literature pertaining to U-Bond™ Ortho is inadequate, and its clinical outcome has not been evaluated to date. The majority of studies on orthodontic adhesive materials have been carried out in *in-vitro* settings, in which obtaining precise simulation of the clinical conditions seems to be an unrealistic goal, taking into consideration various factors associated with an *in-vivo* environment. Hence, to obtain clinically relevant results, the present *in-vivo* study was carried out to evaluate and compare the bonding failure rate of U-Bond™ Ortho with Transbond™ XT, along with a comparative evaluation of the mean bracket survival time with the two orthodontic adhesives. 

The null hypothesis for the study was that there would be no differences in bonding failure rate and mean bracket survival time of U-Bond™ Ortho and Transbond™ XT adhesive materials.

## MATERIAL AND METHODS

The present cross-arch split-mouth prospective study was conducted in the Department of Orthodontics and Dentofacial Orthopedics of a tertiary dental care teaching institution, following ethical clearance obtained from the institutional ethical committee. Standard orthodontic treatment records were taken for the patients reporting to the orthodontic clinic from January 2023 to October 2023, and were screened according to the inclusion criteria, as follows: (a) healthy adolescent/adult patients aged between 15-30 years, (b) complete set of permanent dentition (third molars not included), (c) Class I malocclusion with spacing / crowding < 5 mm in both dental arches, (d) patients with average growth pattern (GoGn-SN being 32±2°), (e) patients requiring fixed mechanotherapy using a nonextraction protocol. Patients were excluded from the study if they: (a) had a history of previous orthodontic treatment, (b) dental anomalies or enamel defects, (c) faulty dental restoration/fixed prosthesis, (d) transverse discrepancy, or (e) skeletal / dental cause of deep bite/open bite interfering with precise bracket positioning.

## SAMPLE SIZE CALCULATION

Each bonded bracket served as the unit of measurement in the present study, hence the sample size was estimated based on the total number of brackets bonded using either Transbond™ XT or U-Bond™ Ortho. Accordingly, a minimum sample size of 660 (330 in each group) was calculated for hypothesis testing with a 95% level of significance and 84% power. Approximately 33 patients were required for the 660 brackets (SupplementaryTable 1). However, to cater for dropouts, 40 patients (20 in each group) were included in the study.

## PROCEDURE METHODOLOGY

Patients who fulfilled the selection criteria were consecutively divided into two groups. In group 1, the first and third quadrants of the dental arches were bonded using the Transbond™ XT adhesive, while the second and fourth quadrants were bonded using the U-bond™ adhesive. In group 2, the first and third quadrants were bonded using the U-Bond™ Ortho adhesive, whereas the second and fourth quadrants were bonded using Transbond™ XT. In both groups, bonding of the maxillary and mandibular arches for each patient was performed by a single operator in a single appointment following standard orthodontic bonding protocols. Fixed mechanotherapy was initiated using a 0.022-in MBT preadjusted Edgewise appliance (OSL signature MBT 0.022” metal bracket, United Kingdom) following standard archwire sequence, and patients were subsequently recalled for observation at 4-6 weeks interval for a total duration of six months. Verbal and written instructions pertaining to diet and dental care were provided to patients at every appointment. 

The following clinical data were obtained for each patient: (a) date of bonding and periodic visit and (b) date and site of bonding failure (if any). In the case of bonding failure, the bracket was not re-evaluated after re-bonding. The mean bracket survival time was calculated as the difference between the date of initial bonding and the date of bonding failure. The date of the periodic visit was taken as the date of bonding failure, in case of patient ignorance. Data were compiled and subjected to statistical analysis.

## STATISTICAL ANALYSIS

All data were statistically analyzed using the Statistical Package for Social Sciences (SPSS, v. 24.0, IBM Corporation, USA) for MS Windows, and the intergroup statistical comparison of the distribution of categorical variables was tested using the chi-square test or Fisher’s exact probability test if more than 20% of the cells had an expected frequency of less than 5. The intergroup statistical comparison of the means of normally distributed continuous variables was performed using an independent sample t-test. Kaplan-Meier survival plot was constructed to perform an intergroup comparison of the incidence of bonding failure. The underlying normality assumption was tested before subjecting the study variables to a t-test. In the entire study, p-values less than 0.05 were considered to be statistically significant.

## RESULTS

Amongst the 40 patients initially included in the study, 4 patients i.e., 2 in each group, were lost to follow-up; hence, 18 patients (Table 1), comprising 360 brackets in each group (Table 2), were included in the final analysis. The bonding failure rate and mean bracket survival time with both adhesive materials were thoroughly studied in the maxillary and mandibular arch, as well as anterior and posterior dentition, along with gender-based distribution of site and frequency of bonding failure. 

**Table 1:** Demographic distribution of sample.

Gender		Cases	Age distribution
Male (n=16)	Group 1	7	15 - 30 years
	Group 2	9	
Female (n=20)	Group 1	11	15 - 28 years
	Group 2	9	
Total		36	Mean age: 22.44 years

**Table 2:** Distribution of data variables.

Groups	Maxillary dentition		Mandibular dentition		Total teeth
	Anterior	Posterior	Anterior	Posterior	
Group 1 (n=18)					
Transbond™ XT	54	36	54	36	180
U-Bond™ Ortho	54	36	54	36	180
Group 2 (n=18)					
Transbond™ XT	54	36	54	36	180
U-Bond™ Ortho	54	36	54	36	180
Total (n=36)	216	144	216	144	720

**Figure 1:**
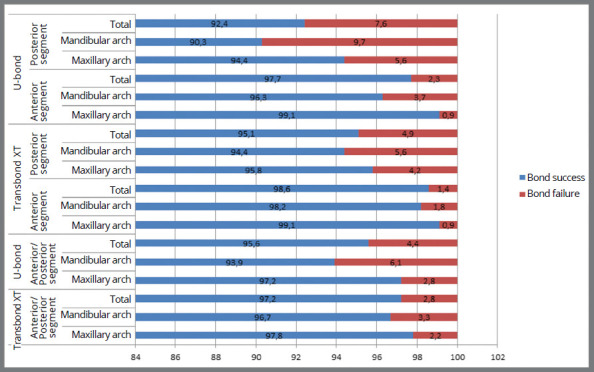
Intergroup comparison of incidence of bonding failure.

A total of 26 (3.6%) bonding failures occurred, being 10 (2.8%) with Transbond™ XT and 16 (4.4%) with U-bond (Table 3). The failure rates of Transbond™ XT were 2.2% and 3.3% in the maxillary and mandibular arches, and 1.4% and 4.9% in the anterior and posterior segments, respectively; while the bonding failure rates with U-Bond™ Ortho were 2.8% and 6.1% in the maxillary and mandibular arches, and 2.3% and 7.6% in the anterior and posterior segments, respectively. All the above intra- and intergroup analysis were found to be statistically non-significant (Tables 4 and 5, Fig. 1).

**Table 3:** Comparative evaluation of overall bonding failure rate between Transbond™ XT and U-Bond™ Ortho.

Adhesive material	Overall result				Total	
	Bond success		Bond failure			
	n	%	n	%	n	%
Transbond™ XT	350/360	97.2	10/360	2.8	360	100
U-bond™ Ortho	344/360	95.6	16/360	4.4	360	100
Total	694/720	96.4	26/720	3.6	720	100
Bond failure rate (Transbond™ XT vs U-Bond™ Ortho)						
P-value: 0.231^NS^						

**Table 4:** Comparative evaluation of bonding failure rate in maxillary and mandibular arches.

Adhesive Material		Overall result			Total			Maxillary arch Vs Mandibular arch
		Bond success		Bond failure				
		n	%	n	%	n	%	
Transbond™ XT	Maxillary arch	176/180	97.8	4/180	2.2	180	100	P-value: 0.750^NS^
	Mandibular arch	174/180	96.7	6/180	3.3	180	100	
	Total	350 / 360	97.2	10 / 360	2.8	360	100	
U-Bond™ Ortho	Maxillary arch	175/180	97.2	5/180	2.8	180	100	P-value: 0.125^NS^
	Mandibular arch	169/180	93.9	11/180	6.1	180	100	
	Total	344 / 360	95.6	16 / 360	4.4	360	100	
Maxillary arch (Transbond™ XT vs U-Bond™ Ortho)				Mandibular arch (Transbond™ XT vs U-Bond™ Ortho)				
P-value: 0.999^NS^				P-value: 0.320^NS^				

**Table 5:** Comparative evaluation of bonding failure rate in anterior and posterior segments.

Adhesive material			Overall result				Total		Anterior vs Posterior segment
			Bond success		Bond failure				
			n	%	n	%	n	%	
Transbond™XT	Anterior segment	Maxillary arch	107/108	99.1	1/108	0.9	108	100	P-value: 0.999^NS^
		Mandibular arch	106/108	98.2	2/108	1.8	108	100	
		Total	213/216	98.6	3/216	1.4	216	100	
	Posterior segment	Maxillary arch	69/72	95.8	3/72	4.2	72	100	P-value: 0.999^NS^
		Mandibular arch	68/72	94.4	4/72	5.6	72	100	
		Total	137/144	95.1	7/144	4.9	144	100	
U-Bond™ Ortho	Anterior segment	Maxillary arch	107/108	99.1	1/108	0.9	108	100	P-value: 0.369^NS^
		Mandibular arch	104 /108	96.3	4/108	3.7	108	100	
		Total	211/216	97.7	5/216	2.3	216	100	
	Posterior segment	Maxillary arch	68/72	94.4	4/72	5.6	72	100	P-value: 0.532^NS^
		Mandibular arch	65/72	90.3	7/72	9.7	72	100	
		Total	133/144	92.4	11/144	7.6	144	100	
Anterior segment (Transbond™ XT vs U-Bond™ Ortho)					Posterior segment (Transbond™ XT vs U-Bond™ Ortho)				
P-value: 0.724^NS^					P-value: 0.330^NS^				

**Figure 2:**
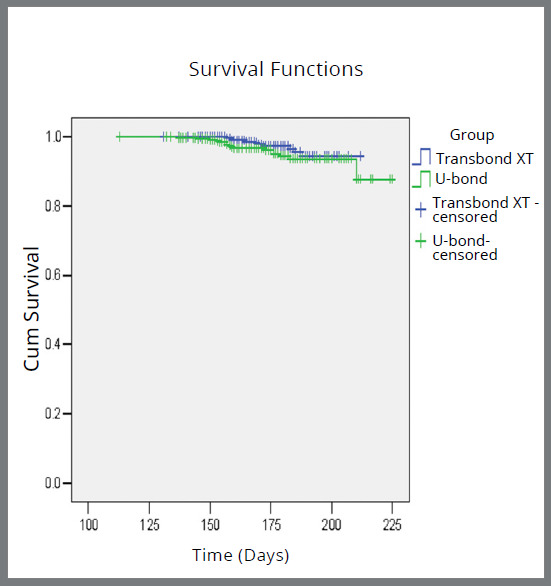
Overall Kaplan-Meier survival plot comparing the incidence of bonding failure.

The mean bracket survival time was 176.25±26.27 days and 173.3± 24.44 days with Transbond™ XT and U-Bond™ Ortho, respectively (Table 6, Fig. 2). With Transbond™ XT, the estimated bracket survival time was 177±31.46 days and 175.5±25.13 days in maxillary and mandibular arches, and 178±31.87 days and 173±24.81 days in anterior and posterior segments, respectively; while the mean bracket survival time with U-Bond™ Ortho was 175.8±29.19 days and 170.8±23.67 in maxillary and mandibular arches, and 176.6±30.13 days and 173.3±23.68 days in anterior and posterior segments, respectively. The intra- and intergroup differences were statistically insignificant (Tables 7 and 8).

**Table 6:** Comparative evaluation of mean bracket survival time (days) between Transbond™ XT and U-Bond™ Ortho.

Adhesive material	n	Mean survival time (days)	P-value
Transbond™ XT	360	176.25± 26.27	0.545^NS^
U-Bond™ Ortho	360	173.33 ± 24.44	

**Table 7:** Comparative evaluation of mean bracket survival time (days) in maxillary and mandibular arches.

Adhesive material	Arch	n	Mean survival time (days)	Maxillary vs Mandibular arch
Transbond™ XT	Maxilla	180	177 ± 31.46	P-value: 0.209^NS^
	Mandible	180	175.5 ± 25.13	
U-Bond™ Ortho	Maxilla	180	175.8 ± 29.19	P-value: 0.641^NS^
	Mandible	180	170.8 ± 23.67	
Mean bracket survival rate in maxillary arch (Transbond™ XT vs U-Bond™ Ortho)			Mean bracket survival rate in mandibular arch (Transbond™ XT vs U-Bond™ Ortho)	
P-value: 0.509^NS^			P-value: 0.166^NS^	

**Table 8:** Comparative evaluation of mean bracket survival time (days) in anterior and posterior segments.

Adhesive material	Arch	n	Mean survival time (days)	Anterior vs posterior segment
Transbond™ XT	Anterior	216	178 ± 31.87	P-value: 0.304^NS^
	posterior	144	173 ± 24.81	
U-Bond™ Ortho	Anterior	216	176.6 ± 30.13	P-value: 0.402^NS^
	posterior	144	173.3 ± 23.68	
Mean bracket survival rate in anterior segment (Transbond XT vs U-Bond)			Mean bracket survival rate in posterior segment (Transbond XT vs U-Bond)	
P-value: 0.611^NS^			P-value: 0.883^NS^	

Among the 26 bracket failures, 15 (57.7%) occurred in males and 11 (42.3%) in females; this difference was statistically significant (p = 0.022) (Table 9). The maximum frequency of bonding failure occurred in the mandibular arch, with the left side being more affected than the right. The mandibular second premolar in the third quadrant was the most affected tooth.

**Table 9:** Gender based distribution of site and frequency of bonded bracket failure.

Adhesive		Maxillary arch (Tooth notation)										Mandibular arch (Tooth notation)										Total bracket failure		
		15	14	13	12	11	21	22	23	24	25	45	44	43	42	41	31	32	33	34	35	Males	Females	
Transbond™ XT	Male	-	-	-	-	-	-	1	-	1	1	1	-	-	-	-	1	-	-	-	1	6	4	Males vs females
	Female	-	-	-	-	-	-	-	-	-	1	-	-	-	-	1	-	-	-	-	2			
U-Bond™ Ortho	Male	-	-	-	-	-	-	1	-	-	2	-	1	-	-	-	1	1	-	1	2	9	7	
	Female	1	-	-	-	-	-	-	-	-	1	1	-	-	-	1	-	1	-	1	1			
Frequency		1	-	-	-	-	-	2	-	1	5	2	1	-	-	2	2	2	-	2	6	15 (57.7%)	11 (42.3%)	P-value: 0.022*
		1					8					5					12					26		
		9										17												

## DISCUSSION

Orthodontic bonding serves as a critical factor in the success of fixed orthodontic mechanotherapy, since frequent bonding failure may result in increased chair-side time, treatment cost, and duration of treatment, which may be frustrating to the patient and the clinician as well.^5^ ^,^ ^6^Further, the re-bonding procedure may result in an enhanced risk of possible damage to the enamel surface.^7^ The success of the bonding procedure depends largely on the characteristics of the bonding agent, although proper isolation and moisture control is required to ensure optimum bonding strength.^8^ Transbond™ XT has been the most successful and thus popularly used orthodontic adhesive for decades; hence, orthodontic bonding with Transbond™ XT was used in the present study as a control group to evaluate and compare the clinical performance, via bonding failure rate, with the recently used U-bond™ orthodontic adhesive.

Transbond™ XT is a light-cured composite resin adhesive containing bisphenol-A-glycidyl methacrylate (Bis-GMA) and triethylene glycol dimethacrylate (TEGDMA) along with fillers and initiators.^3^On the other hand, U-Bond™ comprises a proprietary blend of dimethacrylates, fillers and initiators.^4^ The bonding procedure with both the adhesive material is similar, involving: enamel etching with 37% ortho-phosphoric acid for 10-15 seconds; followed by rinsing and thorough air drying; application of thin and uniform coating of the respective primer on drying enamel surface; followed by application of the adhesive/bonding agent (Transbond™ XT or U-Bond™ Ortho) to the bracket base, positioning the bracket onto the tooth surface, and removing the adhesive excess around the bracket base; followed by light-curing for 10 seconds with a light curing unit (420 -500 nm, >800 mW/cm^2^), to initiate polymerization.^3^ ^-^ ^4^ The*in-vitro* shear bonding strength of the recently introduced U-Bond™ orthodontic adhesive has been claimed by the manufacturer to be as high as 24.9 MPa^4^, which is comparable to that of various commercially available light cure orthodontic adhesives: 21.32 MPa for Transbond™ XT (3M Unitek), 20.05 MPa for Heliosit Orthodontic™ (Ivoclar Vivadent AG) and 23.36 MPa for Enlight™ (Ormco)^9^, all being much higher than the clinically acceptable bonding strength of 5.9 - 7.8 MPa.^2^ Additionally, numerous techniques have been recommended in the literature to enhance the shear bonding strength of orthodontic brackets being bonded to enamel surface.^10^ ^-^ ^14^

In the present study, there was a total of 26 bonding failures accounting for 3.6% overall bonding failure rate, being 10 (2.8%) with Transbond™ XT and 16 (4.4%) with U-Bond™ Ortho, and the difference was not statistically significant. Clinical studies evaluating various orthodontic adhesive systems have reported bonding failure rates ranging between 4.7-6.6%,^15^ 2.7-3.6%,^16^ 4.5-7.7%,^17^ 6-8%.^18^ The bonding failure rate below 10% is generally considered clinically acceptable.^19^

The present study was carried out for a follow up period of six months, since maximum bonding failure occurs during the initial phase of fixed mechanotherapy, as highlighted by O’Brien et al.^15^ Various patient related risk factors contributing to bonding failure may include tooth morphology and eruption status, chewing pattern, masticatory forces varying with vertical facial types, type of malocclusion and resultant mechanotherapy, culturally influenced dietary habits, internal motivation and gender differences.^20^ ^-^ ^22^ Clinician and armamentarium related factors affecting bonding failure may include non-adherence to bonding protocols, inadequate isolation, type and geometry of brackets, design of bracket base, thickness and type of adhesive, enamel surface treatment, use of sub-standard quality material and failure to motivate the patients.^22^ ^-^ ^25^ Adhesive remnant index (ARI) serves as an useful scale for the assessment of type of bonding failure, through the evaluation of the amount of adhesive remaining on the enamel surface and/or bracket base.^26^ Though bonding failure often occurs as a combination of both cohesive and adhesive failure, greater number of bonding failure has been reported by Henkin et al^27^ to occur at enamel and adhesive interface with different brands of metal brackets. However, the type of bonding failure when using U-Bond™ adhesive material was not evaluated in the present study.

The present study demonstrated non-significant differences in the bonding failure rate using Transbond™ XT and U-Bond™, when comparing the maxillary and mandibular arches, as well as anterior and posterior segments, although mandibular bondings failed relatively more than the maxillary bondings, and posterior bondings failed more than the anterior bondings, with both adhesive materials. Similar findings have been reported by other studies.^15^ ^,^ ^17^ The potential reasons could be the relatively heavy occlusal forces exerted on the mandibular and posterior dentition, difficulty in access, and poor moisture control in the posterior dentition.^8^As a result, the maximum frequency of bonding failure occurred in the mandibular left second premolar. An uneven adhesive thickness due to the increased labial curvature of the premolars might be an additional contributory factor to the increased frequency of bonding failure. The same is also evident from the comparatively greater bracket survival time in the maxillary arch and anterior segment. The significantly lower bonding failure rate in females might be due to the greater internal motivation and comparatively lesser magnitude of masticatory forces, compared to males.

Thus, the present *in-vivo* study highlighted the clinical performance of the recently introduced U-Bond™ adhesive as compared to that of Transbond™ XT. However, the potential for bracket displacement during curing, due to the comparatively higher flowable consistency of U-Bond™, may pose a challenge in certain cases.

### LIMITATIONS OF THE STUDY AND FUTURE RECOMMENDATIONS

The present study compared the clinical performance of U-Bond™ adhesive material with that of the popularly used Transbond™ XT. The subjective visual assessment method used in ARI score may be misleading in clinical settings; hence, the nature of bonding failure was not evaluated in the present *in-vivo* study. Stereomicroscopy or scanning electron microscopy provides precise visualization and thus thorough analysis of ARI; hence, it is recommended to further evaluate the nature of bonding failure when using the U-Bond™ adhesive material. Apart from bonding strength and bracket survival rate, there are numerous characteristics for an adhesive to be considered as ideal, which have not been considered in the present study. Hence, further research and clinical trials with large sample size and longer observation periods are recommended to further explore the mechanical/chemical properties -such as the effect of aging processes, i.e., thermal cycling, chemical leaching potential, and cytotoxicity testing-, along with the feasibility of incorporating nanotechnology to enhance the long-term performance and clinical outcome of the recently introduced U-Bond™ Ortho adhesive material. 

## CONCLUSION

Within the scope of this study, the following conclusions were drawn:

(a) The null hypothesis was accepted, as U-Bond™ Ortho and Transbond™ XT adhesives showed statistically insignificant differences in bonding failure rate and mean bracket survival time.
(b) For the right-handed operator, the maximum frequency of bonding failure occurred on the left side of the dental arch, with the third quadrant being the most affected.
(c) The bonding failure rate was significantly greater in males, as compared to females.

## Supplementary table 1: Sample size calculation.

[Table t1s]

**Table t1s:**

Sample size was determined by using the effect sizes from a pilot study and with the help of following formula:
n(Per Group) = ${\left[\frac{Z_{a/2}\sqrt{2pq}+Z_{\beta }\sqrt{(p1q1+p2q2}}{p1-p2}\right]}^{2}$
p1 = 0.015 (Approximate incidence of bonding failure in Group A (Transbond-XT))
p2 = 0.055 (Approximate incidence of bonding failure in Group B (U-Bond))
q1 = 1 - p1 = 0.985, q2 = 1 - p2 = 0.945
Z_a/2_ = 1.96 (score at 95% confidence interval), Z_β_ = Cut-off value
for Power (1 - β) = 0.8416
n (Per Group) =
((1.96*SQRT(2*0.035*0.965)+0.8416*SQRT(0.015*0.985+0.055*945))/(0.015-0.055))^2
= 330.19 per group
Thus, the minimum sample size required according to this formula is 330 per group (660 total in 2 groups)
